# Filling-In and Suppression of Visual Perception from Context: A Bayesian Account of Perceptual Biases by Contextual Influences

**DOI:** 10.1371/journal.pcbi.0040014

**Published:** 2008-02-15

**Authors:** Li Zhaoping, Li Jingling

**Affiliations:** 1 Department of Computer Science, University College London, United Kingdom; 2 Graduate Institute of Neural and Cognitive Sciences, China Medical University, Taiwan; University College London, United Kingdom

## Abstract

Visual object recognition and sensitivity to image features are largely influenced by contextual inputs. We study influences by contextual bars on the bias to perceive or infer the presence of a target bar, rather than on the sensitivity to image features. Human observers judged from a briefly presented stimulus whether a target bar of a known orientation and shape is present at the center of a display, given a weak or missing input contrast at the target location with or without a context of other bars. Observers are more likely to perceive a target when the context has a weaker rather than stronger contrast. When the context can perceptually group well with the would-be target, weak contrast contextual bars bias the observers to perceive a target relative to the condition without contexts, as if to fill in the target. Meanwhile, high-contrast contextual bars, regardless of whether they group well with the target, bias the observers to perceive no target. A Bayesian model of visual inference is shown to account for the data well, illustrating that the context influences the perception in two ways: (1) biasing observers' prior belief that a target should be present according to visual grouping principles, and (2) biasing observers' internal model of the likely input contrasts caused by a target bar. According to this model, our data suggest that the context does not influence the perceived target contrast despite its influence on the bias to perceive the target's presence, thereby suggesting that cortical areas beyond the primary visual cortex are responsible for the visual inferences.

## Author Summary

We study how visual perception of a target bar can be biased by contextual bars in the image, and how a Bayesian model of object inference can account for the data. Human observers are more likely to perceive a target bar when the contextual contrast, i.e., the luminance difference between the contextual bars and background, is weaker rather than stronger. Relative to the situation without the context, they are biased to perceive the target in a context of weak contrast when the target can perceptually group well with the context, as if the context fills in the target. Meanwhile, they are biased not to perceive the target in a context of strong contrast, as if the context suppresses the perception, regardless of whether it could perceptually group well with the would-be target. The Bayesian model illustrates that the context influences the perception by biasing (1) observers' prior belief that a target should be present and (2) observers' internal model of the likely input contrasts from a target bar. Our data suggest that brain areas beyond the primary visual cortex along the visual pathway are responsible for inferring object causes for input images.

## Introduction

### Background

**Figure 1:**
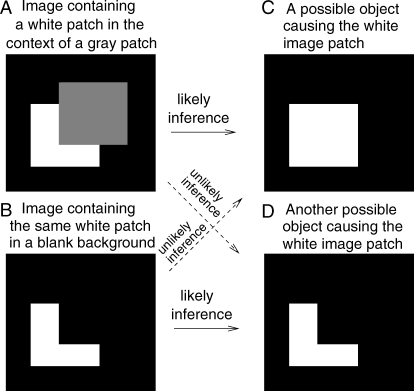
Demonstration of Inferences of Objects from Images (A) and (B) show two images containing the same white patch, and (C) and (D) show the two possible inferred objects in the scene causing this white patch. The inferred causes for any particular input image patch is not unique, although some inferences are more likely than others. The difference in the most likely inferred object for the same image patch in (A) and (B) demonstrates that inference could be greatly influenced by the image context.

Visual inputs are first represented in early visual stages such as retina and the primary visual cortex (V1), such that input features such as local color, orientation, luminance contrast, and spatial scale of image patches are encoded by the activities of retinal and V1 neurons with various input sensitivities. The neural representation of inputs is then used by the brain to infer the possible objects in the 3-D scene causing the 2-D input images. For instance, from V1′s responses to the luminance edges in Figure 1A, the brain could infer a white square surface behind a gray square surface, likely employing cortical area V2 where neurons tuned to surface border ownerships signal which of the possible object surfaces is likely responsible for each luminance edge [1,2]. Information about the object causes are only ambiguously available, or even apparently missing, in the 2-D images. As vision is an under-constrained or ill-posed problem, the possible objects causing a given image are not unique. For instance, the white L-shaped image patch in Figure 1A is likely caused by a white square surface behind the gray one in the 3-D world; but it is not impossible, though less likely, that an L-shaped surface is the cause. Nevertheless, perception is rarely ambiguous, typically revealing only (the most likely) one cause at any time given an input. Here, perception is defined as the result of revealing a cause to visual awareness, while inference is the process of assigning a probability to each cause. As both perception and inference are assessed operationally by the same observer reports, the two words are often used interchangably in this paper. It is difficult to state the veridicality of the perception objectively. For instance, a substantial part of the white square surface (in the 3-D world) is not recorded in the 2-D input image, and would be non-veridical in terms of image pixel values rather than the 3-D world.

Visual inference from any part of the input is often influenced by the contextual input. For instance, the more likely cause for the white patch in Figure 1A or 1B is the square or L-shaped surface respectively, due to the presence or absence of the contextual gray patch. The speed and accuracy to recognize an object, e.g., a sewing machine, significantly depend on, e.g., whether it is in an indoor or outdoor scene [3]; and the color appearance of an image patch depend on the surrounding patches [4]. This is unsurprising since the missing or ambiguous information, e.g., the occluded part of a face or the reflectance of a surface, can only be filled in or deduced from the context through the statistical knowledge about visual scenes, e.g., the correlations between neighboring inputs. Contextual influences are also present in the input encoding. For instance, the sensitivity of a V1 neuron to an input bar can be increased by contextual bars (outside the receptive field of the neuron) aligned with it [5–7], and this colinear facilitation has been manifested in human sensitivity to detect a small bar or gabor (or grating) patch [8–13].

We are interested in contextual influences in inference of objects from images, focusing in this paper on the perception in the spatial context of other inputs. Most previous studies on influences by spatial context used quite complex inputs such as photographs of everyday scenes [14,15], demonstrating very interesting phenomena [16]. However, these complex inputs are difficult to manipulate systematically, and the complex spatial relationships between image features [15] are difficult to describe and model in an intuitive and meaningful way, unless when the exact spatial relationship is not essential such as when inferring surface color appearance [17]. This study uses stimuli that are easy to manipulate and describe. They are composed of several bars, like those used in probing contextual influences on input sensitivity [8–10,12,13].

The previous studies used the stimuli of bars to probe input sensitivities by the two-alternative forced choice (2AFC) design. In contrast, we probe perceptual biases by a yes–no design. In each trial of the 2AFC design, two brief intervals of the stimuli are presented: both intervals contain the same contextual input but only one contains the target, and the observer has to answer which interval contains the target. The input sensitivity is inversely linked with the minimum target input (contrast) necessary to enable about 80% of the responses by the observers to be correct. It has long been known [18] that measurements from the 2AFC tasks remove the effect of any perceptual or response bias (e.g., on whether the target bar is present), whether the bias arises from the contextual inputs or other factors. In each trial of a yes–no task, after only one stimulus presentation interval, observers have to answer “yes” or “no” regarding whether they perceive a target bar, i.e., whether the target rather than noise is the inferred cause of the luminance profile at the would-be target location in the input image. Whether the answer is veridical according to the input images is not the issue; rather, we assess whether the observer *perceives or infers* the target bar, even if its contrast is missing in the input image. This yes–no task thus assesses the bias (to respond “yes”) in inferring the target object. One particular bias is filling-in, which we define as a behavioral indication of a target *object* (by responding “yes”) when there is no input contrast at the corresponding image location. Note that filling-in here is *not* defined as (mentally) painting-in a *luminance contrast* at the image location corresponding to the target object when the input contrast is zero. Analogously, amodel perceptual completion of the occluded square (in Figure 1A) is achieved without seeing any contrast at the image location for the occluded part of the square.

We report in this paper that our study, using the bar stimuli and the yes–no task, revealed how visual contexts influence the perception of the target bar through a Bayesian inference and decision process. In particular, quite unexpectedly from the finding of colinear facilitation of input sensitivities revealed neurophysiologically and behaviorally (by the 2AFC task), we found that weaker colinear contexts induce stronger biases to fill-in the missing target. In the framework of a model of the Bayesian process, our data suggest that contextual facilitation or suppression of input sensitivities plays no role in the inference probed by our task, and hence the neural substrate responsible for this inference is more likely beyond V1. In the rest of the Introduction, we formulate the Bayesian model applied to our yes–no task. The Results section then presents our experiments probing the contextual influences in human inference behavior and the fit of our data by the Bayesian model. The Discussion section will summarize the findings with discussions.

### The Bayesian Model of Contextual Influence on Visual Inference from Simple Bar Stimuli

#### The formulation.

The Bayesian inference and decision process applied to our task is formulated as follows [18,19]. Let a stimulus pattern contain input contrast *C_t_* and *C_c_* for the target and contextual bars respectively, evoking neural responses *x_t_* and *x_c_*, respectively, in the early visual stages. When the target is absent in the image, *C_t_* = 0. For presentation simplicity without loss of generality, the target and context are assumed as sufficiently far apart spatially to evoke dissociable responses. The brain infers from *x_t_* whether the target is present, i.e., whether *x_t_* is caused by the target bar or noise, by assigning a probability *P*(*yes* | *x_t_*) that a target is present given response *x_t_*. By Bayesian theorem, 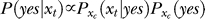 , where 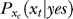 is the probability, by the brain's internal model, of response *x_t_* to a target, and 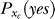 is the prior probability, believed by the brain, that a target should be present. Hence, *P*(*yes* | *x_t_*) is the posterior probability in the Bayesian terminology. Note that 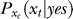 is not a typical likelihood term in Bayesian terminology in which the likelihood typically means the conditional probability of neural response *x_t_* if the experimenter presented a target—instead, 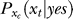 is what the brain thinks the probability of response *x_t_* should be when the brain assumes that *x_t_* is caused by a target, whether or not the experimenter actually presented the target. The subscript *x_c_* in 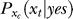 and 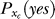 indicates that both could be influenced (or parameterized) by the response *x_c_* to the context. To minimize the mean response error (assumed as the loss function in the decision), the observer's optimal response to the question “is the target present?” is “yes” when *P*(*yes* | *x_t_*) > 0.5 and “no” otherwise. With input and neural noise, the neural responses *x_t_* (and *x_c_*), and consequently *P*(*yes* | *x_t_*) and the observer's response, can vary from one trial to another given a fixed input presentation. Averaged over many trials of a given input image, one can measure the probability *P*(*yes* | *C_t_*) of response “yes” given a target contrast *C_t_* (and context). We can phenomenologically call *P*(*yes* | *C_t_*) the posterior, as the brain's inferred probability of a target being present given the input contrast *C_t_*. It is the counterpart or the manifestation of *P*(*yes* | *x_t_*), internal to the brain and inaccessible to our behavioral measurements. The Appendix section gives a detailed formulation to arrive analogously at the phenomenological internal model *P*(*C_t_* | *yes*) and phenomenological prior *P*(*yes*), the counterparts of 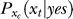 and 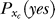 , respectively. For simplicity in the main text, we use this phenomenological language to present the rest of our formulation of the inference process, and omit the details of the decision process (of choosing to respond “yes” or “no” given *P*(*yes* | *x_t_*)) unless it is necessary (e.g., in the Discussion section). To avoid notational clutter, different probabilities, e.g., *P*(*yes*) and *P*(*C_t_* | *yes*), are simply denoted by the differences in the variables, with no or minimum notations for the parameter dependences.

In the Bayesian model, the inferred probability *P*(*yes* | *C_t_*) that *C_t_* is caused by a target bar arise from weighing the two probabilities: one is the probability *P*(*yes*)*P*(*C_t_* | *yes*) that *C_t_* could arise from a target, the other is the probability *P*(*no*)*P*(*C_t_* | *no*) that *C_t_* could arise from “no target” or noise. Here, *P*(*yes*) and *P*(*no*) = 1 − *P*(*yes*) are the prior probabilities, assumed by the brain, of a target as present and absent respectively; and *P*(*C_t_* | *yes*) and *P*(*C_t_* | *no*) are the brain's internal models of the probabilities of having input contrast *C_t_* at the would-be target location when the brain assumes the target is present or absent respectively. Hence,

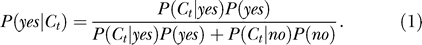



Note that *P*(*yes*), *P*(*no*), *P*(*C_t_* | *yes*), and *P*(*C_t_* | *no*) are the internal belief or models in the observer's brain. In particular, *P*(*yes*) is *not* the probability that the experimenter actually presented a target bar at the target location, nor is *P*(*C_t_* | *yes*) the probability that a contrast *C_t_* is presented at the target location by the experimenter, the “yes” in *P*(*C_t_* | *yes*) refers to the brain's assumed condition of a target present rather than the actual presence of a target placed by the experimenter. Throughout the paper, “yes” and “no” always refer to the observer's responses or internal variables in his/her brain rather than the experimenter's stimulus presentation.

**Figure 2:**
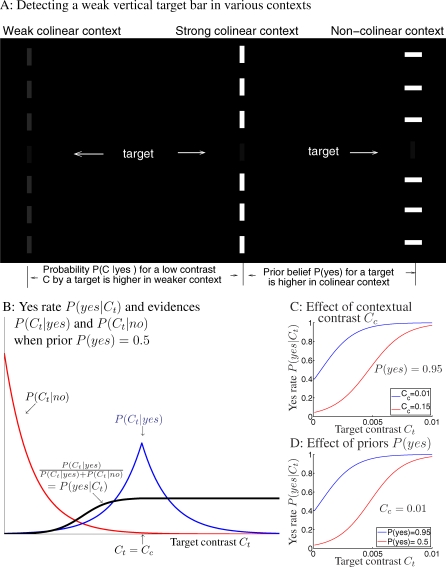
Bayesian Inference for Target Perception (A) Schematics of perceiving a weak vertical target bar in three different contexts. Colinear contexts give a higher prior belief *P*(*yes*) of the target present, as it could be grouped with the context. Higher contextual contrast *C_c_* makes a low contrast input *C_t_* at the would-be target location seem less likely to be caused by a target rather than noise, since observers expect a target to evoke a contrast similar to *C_c_*, i.e., *P*(*C_t_* | *yes*) peaks at *C_t_* ≈ *C_c_*, and *P*(*C_t_* | *yes*) ≈ 0 if *C_t_* ≪ *C_c_*; see (B). (B) the probability *P*(*yes* | *C_t_*) of “yes” response depends on the ratio between the evidences *P*(*C_t_* | *yes*) and *P*(*C_t_* | *no*) for target present and absent, respectively, when the prior belief *P*(*yes*) = 0.5 is unbiased. This ratio should be multiplied by *P*(*yes*)/(1 − *P*(*yes*)) in general. Note that probability distributions *P*(*C_t_* | *yes*) and *P*(*C_t_* | *no*) peak at *C_t_* = *C_c_* and *C_t_* = 0, respectively. (C,D) Effects of the contextual contrast *C_c_* (C) and of the prior *P*(*yes*) (D) by the Bayesian model. In (C) and (D), all curves have model parameters *k* = 2 and *σ_n_* = 0.0015, the two red curves are identical, with *P*(*yes*) = 0.95 and *C_c_* = 0.01. Comparing (C) and (D), a higher contextual contrast *C_c_* has a similar effect as a lower prior *P*(*yes*).

Both *P*(*yes*) and *P*(*C_t_* | *yes*) are subject to observer's biases, which can be influenced by the context, as illustrated in Figure 2. If one occluded from view the target but not the contextual bars, the prior *P*(*yes*) is the observer's expected probability that the target is present behind the occluder. So, *P*(*yes*) is higher in a colinear context, which is seen as more likely to group with target. The context also influences *P*(*C_t_* | *yes*) by making observers expect that the target and contextual bars should have similar contrasts, i.e., the probability *P*(*C_t_* | *yes*) of the target contrast *C_t_* should peak around *C_t_* = *C_c_* (see Figure 2B). We thus have the model

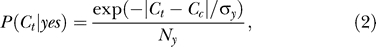
where *σ_y_* models the uncertainty about the target contrast, and 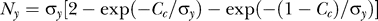 is the normalization constant for the probability distribution on the contrast range 0 ≤ *C_t_* ≤ 1. It is reasonable to assume (see the Appendix section for justifications) that *σ_y_* is proportional to *C_c_* with a Weber-like scale factor *k*,

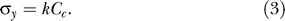



Without the context *P*(*C_t_* | *yes*) is assumed (its exact form does not matter, as it is never fitted to the data) to become 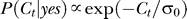 with a contrast uncertainty *σ*_0_. The brain also assumes that input contrast *C_t_* caused by noise or other non-target factors to be near zero; hence,

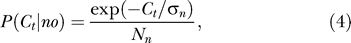
where 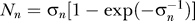 , with contrast uncertainty *σ_n_* determined by the observer's internal model of the noise. From Equations 1–4, we see that three parameters: *P*(*yes*), *k*, and *σ_n_* can completely model *P*(*yes* | *C_t_*) for all *C_c_* and *C_t_*, given a contextual configuration which determines *P*(*yes*).

#### The elaborations.

One may think of *P*(*C_t_* | *yes*) and *P*(*C_t_* | *no*) as evidences for a target present and absent, respectively, and the observer arrives at his response probability *P*(*yes* | *C_t_*) by combining the evidences with his prior belief *P*(*yes*) and *P*(*no*). Both the priors and the evidences are influenced by the context—the prior *P*(*yes*) by the contextual configuration while the evidence *P*(*C_t_* | *yes*) by the resemblance between the contextual contrast *C_c_* and the input contrast *C_t_*. In general, one could model the evidence *P*(*C_t_* | *yes*) and prior *P*(*yes*) such that each could be affected by *both* the configuration *and* the contrast of the context. Insufficient motivation for such a generality, which would nevertheless require additional model parameters, justifies eliminating it by Occam's razor.

Figure 2C illustrates that a higher contextual contrast *C_c_* gives a lower *P*(*yes* | *C_t_*) or suppresses the perception of a target with small *C_t_*, since it makes the low contrast *C_t_* seem as unlikely caused by a target rather than noise. This is because, when the context is clearly visible while the target is barely visible, *C_t_* < *C_c_* (as is always the case in our experiment), the evidence 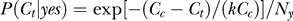 decreases with increasing *C_c_*. In detail, if context one and context two have the same configuration but different contrasts *C_c_*_1_ and *C_c_*_2_ such that *C_c_*_1_ > *C_c_*_2_ > *C_t_*, let *P_c_*_1_ and *P_c_*_2_ denote the probability *P*(*C_t_* | *yes*) under *C_c_*_1_ and *C_c_*_2_ respectively, then, 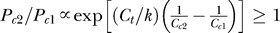 (provided that the normalization constant *N_y_* for *C_c_*_1_ is larger than that for *C_c_*_2_, which is indeed the case for us, as shown in the Appendix section). Meanwhile (see Figure 2D), given a contextual contrast *C_c_* (and thus the evidence *P*(*C_t_* | *yes*)), one is more likely to expect a target in the colinear than non-colinear context since the prior belief *P*(*yes*) is higher in the colinear context.

Figure 2B illustrates that in some ranges of input contrast *C_t_*, the evidences *P*(*C_t_* | *yes*) and *P*(*C_t_* | *no*) for and against a target's presence, respectively, are very different from each other, i.e., 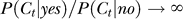 or 0. In such a case, the evidences are unambiguous, diminishing the effect of a prior *P*(*yes*), making the responses (with probability *P*(*yes* | *C_t_*)) also unambiguous. This happens over a large range of small *C_t_* when a stronger contextual contrast *C_c_* pulls the distributions *P*(*C_t_* | *yes*) and *P*(*C_t_* | *no*) apart from each other. When *C_c_* is sufficiently low, there is a sizable range of low input contrast *C_t_* in which the evidences *P*(*C_t_* | *yes*) and *P*(*C_t_* | *no*) for and against a target are comparable, i.e., the evidences are ambiguous, giving the prior *P*(*yes*) the power to sway the response probability *P*(*yes* | *C_t_*).

Filling-in, which occurs when *C_t_* = 0 but *P*(*yes* | *C_t_*) is substantial, is an example when the prior sways the response. It happens particularly when the noise level *σ_n_* is high, such that a zero input contrast *C_t_* could be caused by the target *or* the noise, i.e., *P*(*C_t_* = 0 | *yes*) is non-negligible compared to *P*(*C_t_* = 0 | *no*). The observer's “yes” response when *C_t_* = 0 is analogous to perceiving a white square in Figure 1A without perceiving any luminance contrast at the image location for the occluded corner of the square. For the partially occluded square, perception attributes the missing luminance to the occluder. For the filled-in target bar, perception attributes the zero contrast *C_t_* = 0 to input or neural noise (such as the noise in the photoreceptors or V1 neurons), which causes input contrasts and/or brain responses to fluctuate away from their supposed levels in the noise-free situation. Hence, a “yes” response to zero target contrast, the result of a decision based on a perception (even if vaguely) of the target, is no less veridical than the perception of the partially occluded square. Analogously, one may perceive no target even under non-zero input contrast *C_t_*, when the evidence *P*(*C_t_* | *yes*) for a target is insufficient and *C_t_* is attributed to or explained away by noise, depressing the posterior probability *P*(*yes* | *C_t_*).

The Bayesian inference described above predicts in particular: (1) a weak context encourages filling-in of the visual target object when it is consistent or easily grouped with the target, i.e., *P*(*yes*) is large; (2) a sufficiently strong context can suppress the perception of a weak target since the strong context bias the observer to presume a weak input contrast *C_t_* as caused by noise rather than a target; and (3) the prior belief *P*(*yes*) can be influenced by the spatial configuration of the context in a way that is consistent with the statistical properties of visual inputs. We report experiments confirming the predictions next.

## Results

In the experiments, human observers were asked to answer whether or not they perceive the target by pressing a button. They were informed that the target when present was a nearly visible vertical bar at the center of the fixation array, and that they should make their judgments according to the target alone regardless of the context. We only used naive observers to minimize any systematic bias not related to the contextual stimuli. In each trial, the particular target and contextual (contrast and configuration) condition was unpredictably chosen among all conditions within an experiment.

### Experiment 1: Weaker Contexts Give Higher Yes Rates *P*(*yes* | *C_t_*)

In experiment 1, the context has 10 colinear bars on each side of the target bar (Figure 2A), and its contrast can be one of *C_c_* = 0, 0.01, 0.05, and 0.4, with *C_c_* = 0 for the no context baseline condition. This is to investigate whether weaker and stronger contexts do give higher and lower yes rates *P*(*yes* | *C_t_*) respectively as predicted. Here contrast is defined by Michelson contrast *C* = (*L_max_* − *L_min_*)/(*L_max_* + *L_min_*) where *L_max_* is the luminance of the bar and *L_min_* that of background. Each bar is a rectangle of 0.9° × 0.165° in size, and the centers of the neighboring bars were 1.15° apart. The possible target contrast *C_t_* = 0, 0.002, 0.004, 0.006, and 0.008 span a range from below to somewhat above the typical human contrast detection threshold without context. Each test image was presented for 24 trials for each observer.

**Figure 3:**
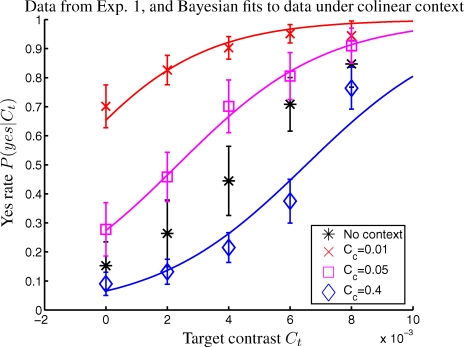
Results from Experiment 1, Where the Colinear Context Resembles the Two Left Ones in Figure 2A The data points are the mean over six observers, and the error bars indicate the standard errors of the means (SEMs). On average and relative to the no-context condition, the weaker colinear contexts *C_c_* = 0.01 and *C_c_* = 0.05 raised the yes rates by CFI = 38% ± 8% and 15% ± 8%, respectively, whereas the stronger context *C_c_* = 0.4 lowered it by −CFI = 17% ± 8%. The colored curves are Bayesian fits to data of the corresponding color, no fit is done for data without context. The root mean square normalized fitting error RMSNFE = 0.66 in the unit of SEM. The fitted parameters (and their 95% confidential intervals) are *k* = 1.9 (0.6, 3.2), *σ_n_* = 0.0025 (0.0020, 0.0029), and *P*(*yes*) = 0.972 (0.967, 0.978).

We found that (Figure 3) compared to the yes rates under no context, the mean yes rates averaged over six observers are higher under low contextual contrast *C_c_* ≤ 0.05 and lower under higher contextual contrast *C_c_* = 0.4, for any target contrast *C_t_*. We define a contextual facilitation index (CFI) as the average increase in the yes rate in a particular context (relative to no context), specifically

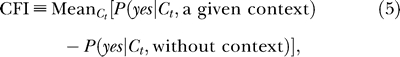
where Mean 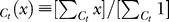 stands for the average of *x* over *C_t_*. The weakest context *C_c_* = 0.01 raises the yes rate by CFI = 0.38 ± 0.08, and the intermediate context *C_c_* = 0.05 by CFI = 0.15 ± 0.08. In contrast, the strongest context *C_c_* = 0.4 *lowers* the yes rate by |CFI| = 0.17 ± 0.08. Averaged over *C_t_*, the observers were more than twice as likely to perceive a target in the weakest than in the strongest context.

The mean yes rates with the context are 86% ± 4%, 63% ± 6%, and 32% ± 5%, respectively, for *C_c_* = 0.01, 0.05, and 0.4 and 48% ± 9% without the context. However, the mean yes rate over trials of all target and contextual conditions is 57% ± 5.5%, suggesting that observers have an internal, stimulus unrelated, prior to roughly equalize their total numbers of “yes” and “no” responses, even though we did not give them any indication of the expected rate of “yes” responses. If the experiment had only one contextual (contrast and configuration) condition, this internal prior could at least partly overwrite the prior caused by the context. Hence, interleaving different contextual conditions within a session helps to manifest and differentiate perceptual biases caused by different contexts.

The adequacy of the Bayesian model is demonstrated by its reasonable fit to the data from the three non-zero contextual contrast conditions, using only three parameters *k*, *σ_n_*, and *P*(*yes*). Let *P_data_*(*yes* | *C_t_*) and *P_fitted_*(*yes* | *C_t_*) be the measured (mean) and fitted yes rates, and *δP_data_*(*yes* | *C_t_*) the SEM error of *P_data_*(*yes* | *C_t_*), and *E* ≡ *P_data_*(*yes* | *C_t_*) − *P_fitted_*(*yes* | *C_t_*) the fitting error. For each data point *i* denoting a particular contextual and target condition, we denote the fitting error and the SEM error as *E_i_* and *δ_i_*, respectively. The quality of the Bayesian fit for a total of *N* data points can be quantified by the root mean squared normalized fitting error defined as

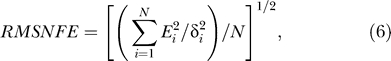
which indicates the fitting error in the units of the SEM errors of the mean yes rates. When RMSNFE < 1, for instance, the fitted curve is within the size of the error bars from the measured data for typical data points. The fitting finds the optimal set of Bayesian model parameters *k*, *σ_n_*, and *P*(*yes*) that minimizes this RMSNFE. Our fit to a total of *N* = 3 × 5 data points for the 3 yes rate curves gives RMSNFE = 0.66. Note that, a psychometric function parameterized by two or more parameters can typically fit a single yes rate curve (which in our case contains five data points). For instance, a logistic function 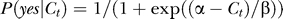 with two parameters, *α* and *β*, could also reasonably fit a yes rate curve in our data. However, three logistic functions or a total of six parameters would be needed to fit three yes rate curves. Hence, fitting our data for three yes rate curves within the error bar by the Bayesian model, using only a total of three parameters, reflects the adequacy of the Bayesian account.

Note that fitting the yes rate data for the no context condition by the Bayesian model would require two additional parameters, *σ*_0_ and the prior probability *P_no context_*(*yes*) under no context, as many as needed by the logistic fit. Hence, fitting this curve well by the Bayesian model adds no additional strength to the Bayesian account. In fact, since the parameter *σ_n_* is already determined from fitting the three yes curves for the colinear context, the two additional Bayesian parameters *σ*_0_ and *P_no context_*(*yes*) are under determined (i.e., many different choices of *σ*_0_ and *P_no context_*(*yes*) would give roughly equally good fits) for a curve that needs only two essential parameters. Thus we display these data as they are without any model fitting.

The higher yes rates under weaker contextual contrasts *C_c_* are not expected from the assumption or expectation that neurons responding to the colinear context should increase the neural response to the target as if the target has an effective contrast 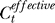 higher than the actual input contrast *C_t_*. If colinear facilitation did make 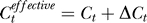 , then the change Δ*C_t_* should depend on the contextual contrast *C_c_* by some function as Δ*C_t_* = *f*(*C_c_*) such that *f*(0) = 0. Then, our Bayesian formulation should replace each *C_t_* in the right-hand side of Equation 1 by 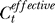 . To the first order (linear) approximation, Δ*C_t_* ≈ *γC_c_*, where *γ* is the coefficient of facilitation. We can then repeat our Bayesian fit with now an additional model parameter *γ*. As expected, this gives a negligible fitted *γ* = −0.5 × 10^−6^ ≈ 0, giving 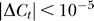 for *C_c_* ≤ 0.4. Hence, no colinear facilitation or suppression of input sensitivities is needed to account for our data, or that our data do not indicate that colinear influence could change the effective contrast of the input.

### Experiment 2: Colinear and Orthogonal Contexts

Experiment 2 was based on Figure 2A, to test that different spatial configurations, one colinear and one orthogonal, of the context can give rise to different prior probabilities *P*(*yes*) according to observers' belief. The colinear context was the same as that in Experiment 1, while the orthogonal context differs from the colinear one only by the orientation of the contextual bars. The contextual contrast used were *C_c_* = 0.01 and 0.4, with another *C_c_* = 0 serving as the no context baseline. Five observers participated in this experiment, each took 20 trials for each condition of a given *C_t_*, *C_c_*, and spatial configuration of the context.

**Figure 4:**
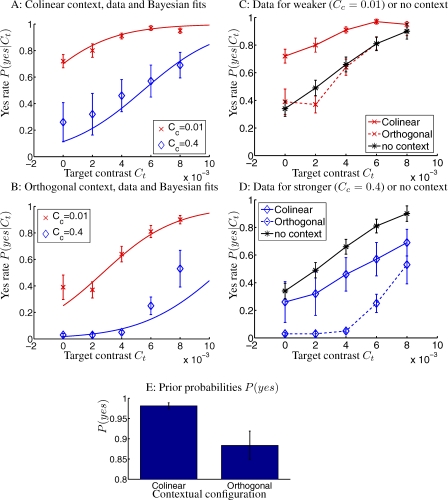
Results from Experiment 2 Averaged Over Five Observers (A,B) Yes rates under colinear and orthogonal context (schematically like Figure 2A), respectively. The curves are the Bayesian fits. The four Bayesian parameters (and their 95% confidence intervals) are *k* = 3.8 (1.8, 5.8), *σ_n_* = 0.0027 (0.0021, 0.0033), *P*(*yes*)*_colinear_* = 0.982 (0.974, 0.989), and *P*(*yes*)*_orthogonal_* = 0.88 (0.85, 0.92), giving a fitting quality of RMSNFE = 1.0. (C,D) Yes rates under different contextual contrast *C_c_* = 0.01 and *C_c_* = 0.4, respectively, together with those under no context. For colinear context CFI = 0.23 ± 0.05 and −0.18 ± 0.15 for *C_c_* = 0.01 and 0.4, respectively; for orthogonal context CFI = −0.018 ± 0.06 and −0.46 ± 0.055 for *C_c_* = 0.01 and 0.4, respectively. (E) Prior *P*(*yes*) for the two contextual configurations. The error bars denote SEMs in (A–D), and 95% confidence intervals in (E).

Figure 4 shows the results. Regardless of the contextual configuration, the yes rate is higher when the contextual contrast *C_c_* is lower, CFI (*C_c_* = 0.01) − CFI (*C_c_* = 0.4) > ≈ 0.4, and a sufficiently high *C_c_* gives negative CFI, biasing the observers to respond “no.” For every contextual contrast *C_c_*, the colinear context gives a higher yes rate than the orthogonal one, CFI(colinear) − CFI(orthogonal) > ≈ 0.23. At low contextual contrast *C_c_*, the colinear context biases the response to “yes” (CFI > 0), while the orthogonal context gives no significant bias. These findings are consistent with our qualitative arguments in Figure 2.

The data can be fitted by the Bayesian model for the four yes rate curves (two configurations × two contextual contrasts) using only four parameters: *k*, *σ_n_*, and the prior probabilities *P*(*yes*)*_colinear_* and *P*(*yes*)*_orthogonal_*, with each data point typically about one error bar size away from the model fit. As expected, *P*(*yes*)*_colinear_* > *P*(*yes*)*_orthogonal_* (Figure 4E). However, both *P*(*yes*)*_colinear_* and *P*(*yes*)*_orthogonal_* are quite high. This we believe is the net result of combining two factors, one is the observers' internal prior to reach roughly equal numbers of “yes” and “no” responses, and the other is the contextual dependent priors from the statistical knowledge of the natural visual environment. Indeed, the average yes rate (over all trials and observers) is 57% ± 2%. The difference between the fitted *P*(*yes*)*_colinear_* and *P*(*yes*)*_orthogonal_* reflects the difference between the natural priors that has survived observers' internal prior imposed by the unnatural laboratory experiment.

### Experiment 3: Different Configurations of Colinear Context

**Figure 5:**
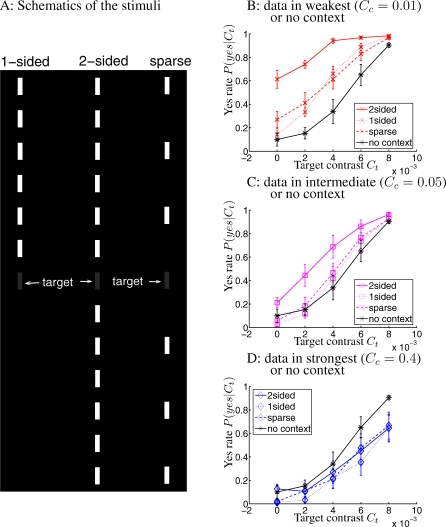
Stimuli (Schematics) and Data for Experiment 3. (A) The schematics of the stimuli. (B–D) Yes rates (with SEM error bars) averaged over seven observers. The 2-sided context gives higher yes rates than other contexts for *C_c_* = 0.01 (B) and *C_c_* = 0.05 (C), but not significantly for *C_c_* = 0.4 (D) when yes rates are all depressed relative to those under no context. The yes rates given a contextual configuration decrease with increasing *C_c_*. Error bars indicate SEM. CFI under the 2-sided, 1-sided, and sparse contexts are respectively: CFI = 0.42 ± 0.06, 0.17 ± 0.04, and 0.19 ± 0.04, for *C_c_* = 0.01, CFI = 0.204 ± 0.06, 0.016 ± 0.07, and 0.05 ± 0.06 for *C_c_* = 0.05, and CFI = −0.11 ± 0.07, −0.18 ± 0.05, and −0.13 ± 0.06 for *C_c_* = 0.4.

Experiment 3 shows that even subtle differences in contextual configuration can manifest in different biases in inferences in ways consistent with the Bayesian account. It is like Experiment 2, but with three colinear context: one is 2-sided which is the one in Experiment 1, removing contextual bars from one end of the target gives the 1-sided context, while removing every alternate contextual bar gives the sparce context, see Figure 5A. The non-zero contextual contrasts are *C_c_* = 0.01, 0.05, and 0.4. Each of the seven new observers took three sessions of data to perform a total of 27 trials for each context condition and *C_t_*.

**Figure 6:**
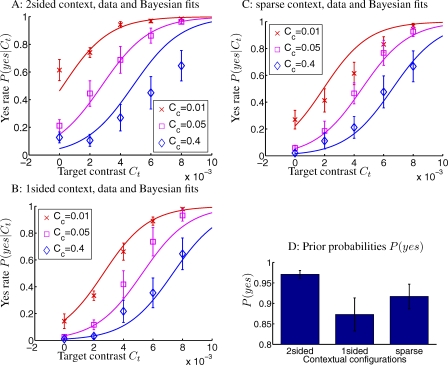
Fit to Data in Experiment 3 by the Bayesian Model (A–C) The red, magenta, and blue curves and data points indicate respective quantities associated with different contextual contrasts *C_c_* = 0.01, 0.05, and 0.4, respectively. The fitted Bayesian parameters (and their 95% confidential intervals) are *k* = 3.91 (1.95, 5.87), *σ_n_* = 1.60 × 10^−3^ (1.45 × 10^−3^, 1.73 × 10^−3^), and *P*(*yes*) = 0.97 (0.96, 0.98) for the 2-sided, *P*(*yes*) = 0.87 (0.83, 0.91) for the 1-sided, and *P*(*yes*) = 0.92 (0.89, 0.95) for the sparse context. RMSNFE = 1.07. (D) The prior *P*(*yes*) for the three different contextual configurations. The error bars denote SEMs in (A–C), and 95% confidence intervals in (D).

Figure 5B–5D show that, the yes rates in the three contextual configurations are very similar for high contextual contrast *C_c_* = 0.4, but the 2-sided context gives the highest yes rates under lower *C_c_* = 0.01 and 0.05, having CFI values about 0.2 higher than those in other contexts. This is consistent with the expectation that the 2-sided context should have the highest prior, and that the subtler differences between the configurations are more easily manifested under lower *C_c_* conditions when observers rely more on the priors for their decisions. Meanwhile, as in Experiments 1 and 2, yes rates decrease with increasing *C_c_* in all contextual configurations. Figure 6 demonstrates that the data in the nine yes rate curves for the non-zero contexts in this experiment can be reasonably well fitted by the Bayesian model using only 5 parameters—*k*, *σ_n_*, and the three *P*(*yes*) values for the three contextual configurations. The *P*(*yes*) for the 2-sided context is indeed the highest, even though, as in Experiment 2, the differences between the three *P*(*yes*)'s must be reduced, by the observers' internal prior, from the true differences between the natural priors.

## Discussion

### Summary of Results

Using simple visual stimuli of bars familiar in psychophysical and physiological studies of *input sensitivities*, our study is one of the first to investigate how visual context bias the *perception* of such visual inputs. In particular, the perception is of the presence or absence of a target bar of a known orientation and shape at a central location given a low or zero input contrast at this location, in the context of other input bar stimuli. We showed that high contrast contextual bars bias the observers to perceive no target bars, as if the context suppresses the perception of the target. Meanwhile, low contrast contextual bars aligned with the target bar bias the observers to perceive a target bar, even when there is zero target contrast in the input image, as if the context fills in the target. This filling-in bias is stronger when the contextual bars have weaker contrasts, and when the target is seen as more likely to group with the context as a straight line.

We show additionally that these findings, unexpected from previous findings of contextual facilitation on input sensitivities, can be accounted for by a Bayesian inference and decision model. The model assumes that the perception results from an inference of the posterior probability 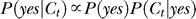 from the following factors: (1) a context dependent prior belief of probability *P*(*yes*) and *P*(*no*) = 1 − *P*(*yes*) of possible visual events “yes” and “no” regarding the target's presence, (2) a (noisy) observation of visual input (contrast) *C_t_*, and (3) the brain's internal model of the context dependent probability *P*(*C_t_* | *yes*) or *P*(*C_t_* | *no*) of the *C_t_* that could be caused by a target or noise. A context that can be better grouped with the target leads to a stronger prior belief *P*(*yes*) of a target's presence. A weak or even zero input contrast *C_t_* is a more plausible evidence for a target (*P*(*C_t_* | *yes*) ≫ 0) in a weaker contextual contrast *C_c_*, since the target is also expected to have a low contrast. In such a case, since evidence *P*(*C_t_* | *no*) for *C_t_* as caused by noise is also non-negligible, the input signal-to-noise is often insufficient to dictate the inference, making the inferred probability *P*(*yes* | *C_t_*) easily swayed by the prior *P*(*yes*). This leads to filling-in when input contrast *C_t_* = 0 but inferred probability *P*(*yes* | *C_t_*) for the target is substantial. In contrast, a high contrast of the contextual bars makes a weak input contrast *C_t_* as seem unlikely caused by a target rather than noise, i.e., *P*(*C_t_* | *yes*) ≈ 0, suppressing the perception of target, i.e., *P*(*yes* | *C_t_*) ≈ 0, even with a large prior belief *P*(*yes*).

### Relating to Previous Studies

The filling-in and suppression of the target respectively in our study is not unlike the visual assimilation and contrast respectively in the perception of brightness [20], color [21,22], tilt [23], or motion direction [24], when the contextual features (brightness, color, tilt, motion) make the target feature appear to shift, respectively, towards or away from the contextual feature. At least in the motion perception, there is also a similar correlation between motion capture versus motion contrast (or induction), analogous to our filling-in versus suppression, and the low versus high signal-to-noise of inputs [24]. In the image encoding process before object inference, there is a similar relationship between the shape of the receptive fields and the signal-to-noise in input—when the input noise is high, the receptive fields of the retinal ganglion cells are large and not spatially opponent, leading to input smoothing which is similar to assimilation; when the input noise is low, the receptive fields have the center-surround spatially opponent shape to enhance input contrast. Such a strategy at the input encoding stage has been understood computationally by efficient coding of visual input information [25,26].

The findings in higher level vision [3,14,15,27,28] that consistent context can facilitate or speed up object recognition or attentional guidance is analogous to our finding that contexts that can be more easily grouped with the would-be target is more conducive to filling-in, reflecting an inference based on information redundancy or correlations in natural scenes. Analogous phenomena of perceptual completion from context are also ubiquitous in mid-level vision [29], including the completion of the missing or incomplete information on object surface color [4], and on occluded or unoccluded surface boundaries [30].

Compared with most of the previous studies on the influences by the spatial context, our study uses simpler stimuli that can be more easily or quantitatively manipulated and described. Consequently, we not only model our data using a simple Bayesian inference and decision model, but also use this model to deduce that, at least in inference, the underlying neural mechanisms do not cause contextual facilitation or suppression of input sensitivities observed at the visual encoding stage [6,31]. Some of the previous studies [4,14], using more controlled stimuli, have also shown that human inference is like that of an ideal observer in a Bayesian inference. In these studies, the Bayesian inferences were based on the *known or built in statistics* of visual inputs. In comparison, we model a Bayesian influence *using a model of* the visual input statistics, parameterized by *P*(*yes*), *k*, and *σ_n_*, which we show is *consistent with the Gestalt grouping laws* which in turn is presumably based on the actual statistics of natural visual inputs. Furthermore, since the target input was independent of the context in the stimulus presentation by the experimenter, the observers' context-dependent perception of the target suggests that they did not modify their internal belief or statistical model of the visual world by sampling the recent stimulus inputs for this task.

### Discussions of Various Issues

Context can change sensitivity to input bars (or bar like elements such as gabors) as manifested behaviorally in 2AFC tasks for target detection [8–12], as if the context effectively changes the input contrast. The primary visual cortex has been argued as the neural substrate for such contextual influences [5,6,31]. However, in our yes–no task probing the inference process, the context does not shift the perceived input contrast from the veridical one according to our model, suggesting that either the brain areas receiving inputs from V1 can somehow distinguish between input sensitivities and input contrast (see [13,32] for related findings), or that the yes–no task somehow evokes the brain to turns off the contextual influences on input sensitivites [33,34]. Hence, the neural substrates responsible for visual inference, in particular for associating neural response *x_t_* with the probability *P*(*yes* | *x_t_*) for a target object, may be beyond V1. This is consistent with the physiological finding [35,36] that V2 rather than V1 is more likely responsible for the illusory contours or disparity capture inferred from the contextual inducers [37], analogous to our filled-in target induced by the context. Also consistent with our finding is the observation [38] that neurons in V2 but not V1 respond to illusory brightness of Cornsweet illusion which manifests the inference of surface (but not image) properties, analogous to the inference of a target object but not contrast features in our task. However, our finding does not preclude the possibility that the inference signals being fed back to V1 from higher cortical areas in subsequent or more advanced processes of inferrence [39,40]. Different mechanisms for input discrimination (sensitivity) and object appearance (inference) have also been demonstrated behaviorally in luminance and surface processing [41].

In previous studies of contextual influence on visual inferences, researchers probed perception by asking the observers to report the appearance, e.g., color and motion direction, of the stimuli. Our study may seem different by asking for reports of whether the target is perceived or not, rather than the appearance, e.g., apparent contrast. However, in essence, the question of “whether you perceive the target or not” is not unlike a question “whether the luminance profile at this location appears as if it is caused by a target or by noise,” which probes the appearance of the perception evoked by the input at the image location concerned. If we had instead asked for reports of apparent contrast, these reports may or may not directly reflect the process of *inferring* the underlying *surface objects* causing the contrast; rather, they may instead reflect the process of *encoding* the 2-D *image* property. In a previous study on color matching [42], observers' responses when asked about the hue and saturation of input showed little color constancy, i.e., the responses did not reflect the underlying surface causes; meanwhile, for the same input, when asked about the underlying paper (objects which reflected the color for the input), the responses showed color constancy. We believe that our request to report the target's presence or absence is more like the request to report on the paper object, thus probing inference.

It is in principle possible that the bias in the observers' reports did not arise from the inference stage (which gives *P*(*yes* | *C_t_*), or more strictly, *P*(*yes* | *x_t_*)), but from the subsequent decision stage, when a threshold value *P_th_* is chosen such that a response “yes” or “no” is given if *P*(*yes* | *x_t_*) > *P_th_* or otherwise respectively [43]. The decision bias would be manifested in the choice of *P_th_*, e.g., *P_th_* = 0.5, 0.1, or 0.9. Our experiments can not distinguish between these two types of biases. However, if the bias was indeed only in the decision (in terms of *P_th_*), then the inference *P*(*yes* | *x_t_*) is independent of the context. Without any insight on how contexts bias the decision threshold *P_th_*, the decision bias has to be modelled by introducing one model parameter for each contextual condition (defined by a particular combination of the configuration and contrast *C_c_* of the context), in addition to the model parameters for the unbiased inference *P*(*yes* | *C_t_*) or *P*(*yes* | *x_t_*) shared by all contextual conditions. Hence decision bias is a less parsimonious model to account for our data since it would require more model parameters than our model of inference bias. In addition, other than a numerical value *P_th_*, the decision bias does not give any insight in why and how the decision should be biased by context when the inference is unbiased. It is most likely that our measured yes rate results from the combined effect of (1) a context specific inference bias in the posterior *P*(*yes* | *x_t_*), and (2) a context independent decision bias in *P_th_* arising from observers' wishes to give the “yes” response in roughly half of all trials. As our task can not distinguish between these two biases, our fitted values for *P*(*yes*) manifest the combined effect from both biases, as discussed in the Results section.

One may wonder whether the sensitivities in the 2AFC task could be derived as the derivatives of the psychometric function (the yes rate) observed in our yes–no task using the same stimuli [44]. The answer is not so. First, it is likely, as discussed earlier, that different mechanisms are involved in input discrimination (for assessing sensitivity) and object inference, such that the input sensitivities and yes rates may not be so simply related. The second reason for the negative answer is the following. The 2AFC tasks were typically performed in blocked sessions, each having only a single contextual condition, while our yes–no design randomly interleaves trials of the different contextual conditions, such that observers compensate fewer “yes” responses in one contextual condition by more “yes” responses in another within a single session. Hence, the yes rates in one context is influenced by the other contexts interleaved within the same experimental session. Consequently, the three yes rate curves in the same no context condition in our three experiments are different from each other, and none of them could be simply related to the sensitivites in the 2AFC task performed in blocked trials. Recently, Polat and Sagi [45] also found, by a yes–no design, different biases to respond “yes” for a gabor target in different colinear contexts (in terms of different target-context distances), when trials of different contextual conditions were interleaved. In comparison with their study, the current study additionally reveals how this bias depends on the contextual contrast, how a Bayesian model can explain the data, and our additional data and the model have enabled us to show that there is no colinear facilitation or suppression of target contrast in such a visual inference task.

In our model, the parameters *k* and *σ_n_* reflect the brain's internal model of the sensory world and its encoding. This internal model adapts quickly to the statistics of the external inputs [46], in particular, to the collection of the inputs presented in an experiment. Therefore, our different experiments, using different collections of stimuli, will evoke different internal models, as manifested by the different values of the model parameters *k* and *σ_n_*.

Our observers seemed unconsciously to use prior beliefs induced by context, despite our instructions informing them that the context was irrelevant to the task. Furthermore, they could quickly switch from one prior to another as the context changes from one trial to another. However, these different priors are only different from the perspective of the target alone. When combining target and context as a whole, the joint prior probability of the visual input in principle arises from the same underlying probability distribution [47] of visual inputs derived from the ecological experience of the observers. Combining computational modeling with psychophysical experiments using easily controlled stimuli, the method in this study enables linking the visual inference behavior with plausible neural substrates. The current study is only a beginning of using such a method, which can be a powerful tool in future studies of visual inference processes.

## Materials and Methods

### Stimuli.

The stimuli were shown on a gamma-corrected 21 inch Sony GDM-F520 monitor using 14-bits luminance resolution. The viewing distance was 67.6 cm, and the screen width was 40 centimeters. All stimulus (target or contextual) bars were rectangular shapes of 0.9° × 0.165° in visual angle, with a luminance *L_max_* no smaller than the background luminance of *L_min_* = 15.6 cd/m^2^ such that the contrast of a bar is (*L_max_* − *L_min_*)/(*L_max_* + *L_min_*); the vertical target bar was always at the display center. Pilot experments established that the contrast detection threshold without contexts is around *C_t_* = 0.005, measured in a 2AFC task with the stair case method. The stimuli were always presented with four black discs, of size 0.2° in diameter, at the four corners of an imaginary square centered at the target location, the side of this square is 1° in visual angle. These four black discs alone on the background also served as the fixation stimulus.

### Procedure.

Each observer was between 18–40 years old, had normal or corrected-to-normal vision, and participated in only one experment. The experiments were carried out in a dimly lit room. Each trial began with the fixation display for 500 ms, followed by the test stimulus display for 80 ms together with an auditory beep, which is then followed by the fixation display which stayed on waiting for observers' button press response to indicate whether they perceived the target or not in the trial. No feedbacks were given regarding whether their responses were correct. The next trial started 800 ms after the button press. A total of 20 randomly selected trials were performed before data collection for each observer before each session. Each experimental session randomly interleaved different stimulus conditions, such that the observers could not predict beyond chance the target contrast *C_t_*, nor the contextual configuration and contrast *C_c_* before each trial.

### Appendix. *Formulation of the Bayesian influence and decision.*

Here we formulate our Bayesian inference and decision model in more detail. In a single trial, *x_t_* and *x_c_* are the neural responses to the target and the context respectively. The target stimuli is uniquely described by the target contrast *C_t_*, as its other aspects (orientation, location, etc) are fixed. The contextual input is determined by both its contrast *C_c_* and its spatial configuration *S_c_* (describing orientation and location). Neural and input noise make *x_t_* a random variable according to a conditional probability *P*(*x_t_* | *C_t_*) of *x_t_* given *C_t_*, and similarly, *x_c_* according to *P*(*x_c_* | *C_c_*, *S_c_*). The brain infers whether *x_t_* is caused by a target or noise for the observer to respond “yes” or “no” to the question “is the target present?” This inference is partly based on the brain's internal model, expressed in conditional probability, *P*(*x_t_* | *yes*) or *P*(*x_t_* | *no*), of how likely *x_t_* can be by target or non-target cause, when the brain assumes the target is present or abstract respectively. Contextual influences on the internal model *P*(*x_t_* | *yes*) is indicated by adding a subscript *x_c_*, in 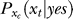 , denoting that *P*(*x_t_* | *yes*) is parameterized by *x_c_* (we assume for simplicity that the context does not influence *P*(*x_t_* | *no*)). The inference is also partly based on the context dependent prior probability 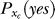 , assumed by the brain, that a target bar should be present. By the Bayesian formula, the brain infers from *x_t_* that the probability for a target to be present in this trial is

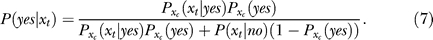



If the observer responds “yes” or “no,” the probability of error is 1 − *P*(*yes* | *x_t_*) or *P*(*yes* | *x_t_*), respectively. To minimize error (assuming that the error rate is the loss function for the decision), the optimal response is “yes” when *P*(*yes* | *x_t_*) > 0.5 and “no” otherwise. Averaging over many trials of fluctuating neural and observer responses, we obtain the probability of “yes” response for a given target and contextual stimuli (*C_t_*, *C_c_*, *S_c_*):

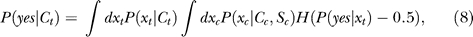
where *H*(.) is a step function such that *H*(*x*) = 1 or 0 when *x* > 0 or otherwise, respectively.

The posterior probability *P*(*yes* | *C_t_*) should depend on *C_t_*, *C_c_*, and *S_c_*, with some functional parameters derived from the functional parameters in 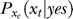 , *P*(*x_t_* | *no*), *P*(*x_t_* | *C_t_*), *P*(*x_c_* | *C_c_*, *S_c_*), and 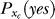 . For our purpose, all we need is to parameterize the dependence of *P*(*yes* | *C_t_*) on *C_t_*, *C_c_*, and *S_c_* by a suitable phenomenological model that has enough parameters, but, applying Occam's razor, not too many. Hence, we use the following Ansatz

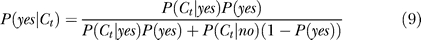
using three phenomenological parameters: one is *P*(*yes*) to parameterize the dependence on *S_c_*, and the other two *σ_n_* and *k*, parameterizing the dependence on *C_c_* and *C_t_*, are defined in the definition of *P*(*C_t_* | *yes*) and *P*(*C_t_* | *no*) as

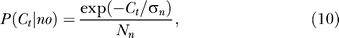


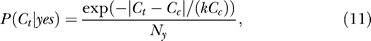
where *N_n_* and *N_y_* are normalization constants such that 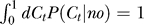 and 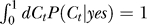 .

While an Ansatz is typically justified by its suitability in accounting for the data, as demonstrated in the main text for the Ansatz above, here we provide some motivations behind this Ansatz. For ease of presentation, we abbreviate the integration 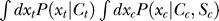 over internal variables by ∮ *d* X℘. Equation 8 suggests an approximation 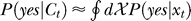 . Then, the certainty equivalent approximation of this equation suggests Equation 9, with approximations 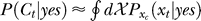 , 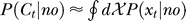 , and 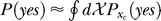 . These approximations do not need to be accurate, since the model parameters are to be fitted by behavioral data rather than derived from integrating these equations. They simply serve to suggest that Equation 9 is a suitable phenomenological model, with *P*(*yes*) the phenomenological prior, and *P*(*C_t_* | *yes*) or *P*(*C_t_* | *no*) the phenomenological conditional probability, assumed by the brain, that the input contrast should be *C_t_* for a target bar or otherwise, respectively.

The model 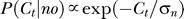 is motivated by the brain's internal model that, without a target, the perceived *C_t_* is more likely zero than another value *C_t_* > 0. Under a simplifying assumption that 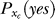 is influenced only by the contextual configuration *S_c_*, 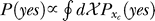 becomes a mere parameter for each contextual configuration. Meanwhile, the form of *P*(*C_t_* | *yes*) is motivated by its approximation 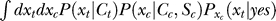 as follows. Physiologically [48,49], the encoding neural response is roughly a sigmoid-like function of the logarithm of input contrast, i.e., *x_c_* = *g*(log*C_c_*)+ noise, with *g*(.)denoting this sigmoid like function. Thus, 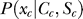 peaks around 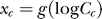 and decreases with 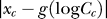 (this is presumably the basis of the Weber law: that the behavorially just discriminable contrast difference between a pedestal contrast and a second contrast is proportional to the pedestal contrast). Similarly, 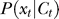 peaks around 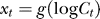 and descreases with 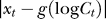 . Assuming again for simplicity that 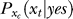 is only influenced by the contextual contrast *C_c_*, the response *x_c_* to a context bar makes the brain expect that *x_t_* should resemble *x_c_* (which are after all examples of neural responses to stimulus bars), making 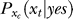 peak around 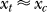 . Combining these observations, 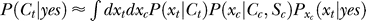 as a function of *C_t_* and *C_c_* should depend approximately on the difference log*C_c_* − log*C_t_* or the ratio *C*_t_/*C_c_*. The model 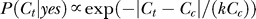 suits such a form, whereas an alternative like 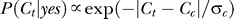 (with a fixed parameter *σ_c_*) would not.

Other additional variabilities, such as the perceived locations of the stimulus, would behave analogously to the internal variables *x_t_* and *x_c_* which should be integrated over, as in Equation 8, to arrive at the experimental observation *P*(*yes* | *C_t_*). One could generalize the definition of *x_t_* and *x_c_*, making each a vector with multiple components for multiple variables, e.g., the first component of *x_t_* for the neural response to the target contrast, the second the neural representation for the target location, etc. Repeating the above derivations would lead us again to Equation 9. By not detailing these additional variables, we are assuming that they will not significantly affect the suitability of our phenomenological model in Equations 9–11. The fitted model parameters manifest the combined effects from all the variables *x_t_* and *x_c_*, even though only a fraction of them play a dominant role.

*Considering contextual influences on the encoding process.* Context could affect the target encoding by changing *P*(*x_t_* | *C_t_*). We consider a situation when context could change input sensitivity such that the encoding neurons respond as if the input contrast is effectively 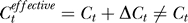 . If *P*(*x_t_* | *C_t_*) without the context takes a functional form *P*(*x_t_* | *C_t_*) = *F*(*x_t_*, *C_t_*) where *F*(.) is some function of *x_t_* and *C_t_*, the contextual influence makes 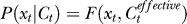 . This motivates the phenomenological formulation to modify the right-hand side of Equation 9 such that *C_t_* is replaced by 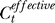 . This contextual influence in encoding can then be phenomenologically modelled by parameterizing the dependence of Δ*C_t_* on the context as, e.g., Δ*C_t_* ≈ *γC_c_*, as done in the main text.

Proof of 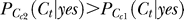 *when C_t_ < C_c2_ < C_c1_ for contextual contrasts C_c1_ and C_c2_ concerned.* We use subscript *C_c_* in 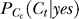 to denote that this probability of target contrast *C_t_* is parameterized by contextual contrast *C_c_*. When 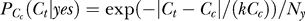 with 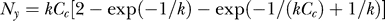 , we have, denoting *N_y_* for *C_c_*_1_ and *C_c_*_2_ as *N_y_*(1) and *N_y_*(2), respectively,

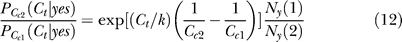



Since 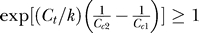 , 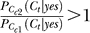 if 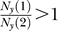 . Note that 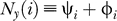 where 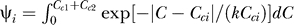 and φ_*i*_ = 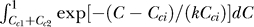 for *i* = 1, 2. Changing integration variable *C* → *C_c_*_1_ + *C_c_*_2_ − *C* in 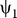 we have 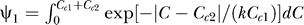 , hence 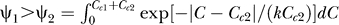 given *C_c_*_1_ > *C_c_*_2_. Meanwhile, 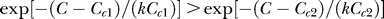 for all *C* ≥ *C_c_*_1_ + *C_c_*_2_. Hence, 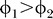 as long as *C_c_*_1_ + *C_c_*_2_ < 1, i.e., the contextual contrasts are not super-saturating. This applies to all of our experimentally used contrasts *C_c_* ≤ 0.4. (In fact, when contextual contrasts are beyond this range, neural responses are saturating and our phenomenological model of the form 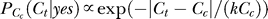 may or may not be the most suitable.) Hence, *N_y_*(1) > *N_y_*(2), and then 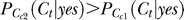 .
